# The utility of a health risk assessment in providing care for a rural free clinic population

**DOI:** 10.1186/1750-4732-1-8

**Published:** 2007-03-23

**Authors:** Paula D Scariati, Cyndy Williams

**Affiliations:** 1 Department of Preventive Medicine, Edward Via Virginia College of Osteopathic Medicine, 2265 Kraft Drive, Blacksburg, VA24060, USA; 2 6308 Rancho Mission Rd. #179, San Diego, CA92108, USA; 3 Virginia Tech Corporate Research Center, 1880 Pratt Drive (0493), Suite 2000, Blacksburg, VA240606, USA

## Abstract

**Background:**

Free clinics are an important part of our country's health safety net, serving a working poor uninsured population. With limited resources and heavily dependent upon volunteer health care providers, these clinics have historically focused on stopgap, band-aid solutions to the population's health problems. Embracing a new paradigm, free clinics are now prioritizing resources for disease prevention and health promotion.

**Methods:**

We initiated a Healthy Friday Clinic project in a rural, southwest Virginia free clinic. The clinic operated every Friday and was open to all people eligible for care in the free clinic. Each participant completed a 43 question Health Risk Appraisal which was used to calculate current risk age (age as determined by current lifestyle choices), optimal risk age (age with optimal lifestyle choices) and potential risk years gained (current risk age - optimal risk age) as well as a ranked listing of modifiable risk factors.

**Results:**

The total sum of potential risk years gained in the free clinic population of 186 subjects was 371.4. Frequency distributions on potential risk years gained by each of the eleven modifiable risk factors revealed the following, in order of impact: quitting smoking could result in a total of 173.5 risk years gained; reducing alcohol consumption, 64.2 years gained; reducing blood pressure, 50.8 years gained; increasing seatbelt use, 38.2 years gained; weight reduction, 24.7 years gained; having regular mammograms, 6.8 years gained; reducing cholesterol levels, 5.8 years gained; reducing frequency of speeding while driving, 3.5 years gained; having regular pap tests, 2.3 years gained; improving HDL levels, 0.9 years gained; and reducing use of smokeless tobacco, 0.8 years gained. Each person received an individualized letter explaining his evaluation along with resources for making changes.

**Discussion:**

Health risk assessments play a role in changing health beliefs and behaviors by providing subjects with individualized feedback on how their lifestyle choices impact their health and well-being. Summed data from health risk appraisals can also be a useful tool in determining the allocation of limited health resources. Whether health risk assessments impact health outcomes directly needs to be studied.

## Background

Over half of all deaths before the age of 65 are attributable to lifestyle factors [1,2]. To reduce the annual incidence of these causes of death, it is essential to understand the contribution of factors such as smoking, smokeless tobacco, alcohol consumption, substance abuse, nutrition, exercise, stress, driving habits, seatbelt usage, and the use of preventive services such as mammograms. The tools that can help assess the impact of these precursors of disease and trauma include the methodology of Health Risk Appraisal (HRA).

HRA is a systematic approach to collecting information from individuals that identifies risk factors, provides individualized feedback, and links the person with at least one intervention to promote health, sustain function and/or prevent disease. A typical HRA instrument obtains information on demographic characteristics (e.g., sex, age), lifestyle (e.g., smoking, exercise, alcohol consumption, diet), personal medical history, and family medical history. In some cases, physiological data (e.g., height, weight, blood pressure, cholesterol levels) are also obtained [3]. The term health risk assessment is sometimes used interchangeably with health risk appraisal. However, Anderson and Staufacker differentiate the two: "...HRA formally refers only to the instrument whereas health risk assessment refers to the overall process (e.g., orientation, screening, interpretation, counseling) in which the HRA instrument is used [4]". Although there is much dialogue about the validity of individual HRAs [5-8], evidence suggests HRA effectiveness when used in a comprehensive worksite health promotion program [9].

Smyth County, one of 39 rural counties in Virginia, covers 452 square miles of the Southwestern tip of the state and sits against the backdrop of the Blue Ridge, Appalachian, and Iron Mountains. The county is comprised of six towns, each with a population less than 6,500, and three public high schools; the county seat is Marion. As of the 2000 Census, the population was 97% white with a population density of 73 persons per square mile [10]. The unemployment rate dropped from 9.9% in June 2004 to 4.5% in November, 2004, the first decrease in three years [11].

According to Virginia vital statistics, the heart disease rate in Smyth County in 2002 was 356.4 per 100,000 population compared to the State rate of 204. Malignant neoplasm rates were 283 per 100,000 population versus 185 for the State, and the rate of diabetes in Smyth County was 27 per 100,000 population where it was only 21 for the State. [12] As with many rural communities [13], these high rates of chronic disease are thought to reflect the older and more disabled population that lives in Smyth. For example, in Virginia 11.2% of the population is 65 years of age or older; whereas 16.8% of the Smyth County population falls into that age category [14]. In addition, this population, like many other rural populations, exhibits poorer health behaviors (i.e., higher rates of smoking and obesity and lower rates of exercise) that are difficult to modify and costly to support.

The Smyth County Free Clinic is a private, nonprofit, community-based organization that is a key provider of health care services in this region. Eligibility criteria for the clinic include being employed, uninsured, and earning an income less than 150% of the federal poverty guidelines [15]. In 2004, 431 new patients enrolled at the free clinic giving it a total patient base of 1750. Patient visits for the same year totaled 2659.

At their inception, free clinics were perceived as stopgap, band-aid solutions that were temporary sources of care until universal health care could be achieved, but that paradigm has since changed. Free clinics are now part of a permanent, integrated health safety net. As such, services are also shifting from predominantly acute medical management toward disease prevention and health promotion.

On February 4, 2005 we launched the Healthy Friday Clinic out of the Smyth County Free Clinic. This new program, staffed by a physician from our osteopathic college, our project manager, and the regular free clinic staff, provided continuity of care for patients with complex health needs as well as comprehensive Health Risk Appraisals (HRAs) for all participants. The Institutional Review Board at the Virginia College of Osteopathic Medicine approved this project on February 1, 2005 as part of a larger community-based pilot project.

## Methods

On February 1, 2005 we held a press conference at the Smyth County Community Foundation to announce our new community-based health project. The conference was well-attended by residents, the local press, and community leaders. An article in the local newspaper and a report on the evening news were useful in creating a heightened awareness of this new project.

That Friday, when we launched the Healthy Friday Clinic, interest was high. People came to see the doctor and to better understand this new partnership between the local free clinic and an osteopathic medical college nearly 70 miles away. We explained our clinic was focused on providing continuity of care for eligible patients with complex health needs, screening for modifiable risk factors, and assessing community issues such as contaminated well-water supplies. Subjects enrolled by completing an informed consent and Health Insurance Portability and Accountability Act (HIPAA) form. They were then invited to complete an HRA and water evaluation form.

The Midlife Healthier People Network Health Risk Appraisal Questionnaire and processing software were provided to us at no charge by the Healthier People Network [16]. This particular HRA tool was an outgrowth of projects done at the Centers for Disease Control and Prevention and the Carter Center of Emory University in Atlanta between 1985 and 1991. In 1991, The Healthier People Network was established as a freestanding 501-c-3 non-profit corporation to ensure the long-term viability of a public interest health risk appraisal program. The Healthier People Network continues the tradition of updating the science underlying the HRA, enhancing the technology to facilitate its use, and broadly disseminating it so public interest can be served.

**Figure 1:**
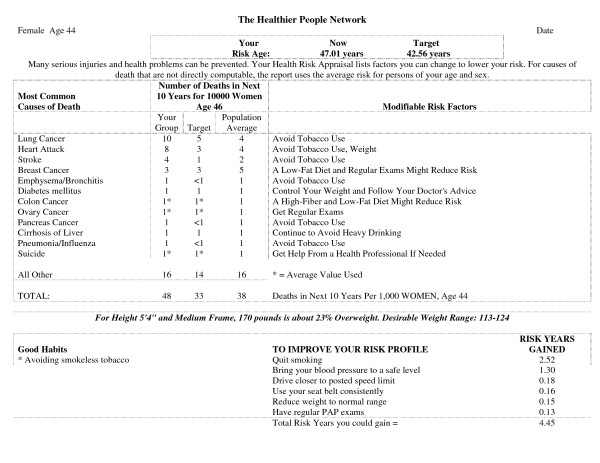
Example Health Risk Appraisal Output from the Healthier People Network Health Risk Appraisal.

This HRA is composed of 43 questions and utilizes algorithms for 19 different causes of death to produce an output that calculates current risk age (age as determined by current lifestyle choices), optimal risk age (age with optimal lifestyle choices) and a list of the modifiable risk factors and their impact on the difference between the current risk age and optimal risk age (Figure 1).

For example, a 44 year old female filling out her HRA notes that she is smoker who has high blood pressure, drives over the speed limit, hasn't had a PAP exam in over 3 years, eats a high fat diet but exercises at least 3 times per week. She is 5 feet 4 inches tall, weighs 170 pounds and estimates herself to have a medium body frame. Her printout shows a current risk age of 47.01 and an optimized risk age of 42.56. The difference between these two measurements (current risk age - optimized risk age) is the potential risks years that could be gained through lifestyle modification. The printout further quantifies that the patient in this example could gain 4.45 years total through a combination of smoking cessation (2.52 risk years gained), better blood pressure management (1.30 risk years gained), driving closer to the posted speed limit (0.18 risk years gained), using her seatbelt consistently (0.16 risk years gained), reducing her weight into a normal range (0.15 risk years gained), and having a PAP exam on a regular basis (0.13 risk years gained). Her good habit of avoiding smokeless tobacco is also recognized and congratulated.

Because the format of the printout was difficult for our patient population to understand and interpret, our project coordinator took the HRA output and generated a personal letter for each participant. The output was simplified and explained in detail. Most letters were 2 to 3 pages in length. We also enclosed educational materials and information on appropriate community resources.

To better understand which modifiable risk factors were having the greatest impact on this free clinic population, we ran frequency distributions on all the risk factors contributing to potential risk years gained. After adding them together to create a denominator, we looked at the attributable portion of each risk factor to the total number of potential risk years gained.

All data were double entered and cleaned in Microsoft Excel™. Analyses were performed using Statistical Package for the Social Sciences version 14.0 (SPSS, Inc., Chicago, Illinois).

## Results

Between February 4, 2005 and December 30, 2005, 299 new patients were seen in the Healthy Friday Clinic. Two hundred and twenty-one of these patients (73.9%) enrolled in the project, meaning they completed an informed consent and HIPAA release. Each participant was also encouraged (but not required) to complete the Healthier People's Network HRA [15] and a water evaluation form. The remainder of this section will focus on the 186 participants who completed the HRA.

Of the 186 participants, 119 (64.0%) were female; 67 (36.0%) were male. The population was predominantly white (n = 178, 96%) and the mean age of the population was 37.7 years; the mode was 34 years. The majority of participants (n = 61, 32.8%) were between the ages of 31–40 years; and 84 (45.2%) had a high school education (Table 1).

**Table 1:** Select Characteristics of Patients Attending the Healthy Friday Clinic, February – December 2005

		**Number of Males (%)**	**Number of Females (%)**	**Total (%)**
Gender		67 (36.0)	119 (64.0)	186
				
Age	18–30	19 (28.4)	35 (29.4)	54 (29.0)
	31–40	24 (35.8)	37 (31.1)	61 (32.8)
	41–50	17 (25.4)	27 (22.7)	44 (23.7)
	51–60	7 (10.4)	19 (16.0)	26 (14.0)
	61+	0 (0)	1 (0.8)	1 (0.5)
				
Race	White	66 (98.5)	112 (94.1)	178 (95.7)
	Black	0 (0)	3 (2.5)	3 (1.6)
	Asian	0 (0)	1 (0.8)	1 (0.5)
	Native American	0 (0)	0 (0)	0 (0)
	Other	1 (1.5)	2 (1.7)	3 (1.6)
	Unknown	0 (0)	1 (0.8)	1 (0.5)
				
Education	< High School	23 (34.3)	22 (18.5)	45 (24.2)
	High School	30 (44.8)	54 (45.4)	84 (45.2)
	Some College	9 (13.4)	32 (26.9)	41 (22.0)
	College Graduate	5 (9.0)	9 (7.6)	14 (7.5)
	Post Graduate	0 (0)	1 (0.8)	1 (0.5)
	Unknown	0 (0)	1 (0.8)	1 (0.5)

**Figure 2:**
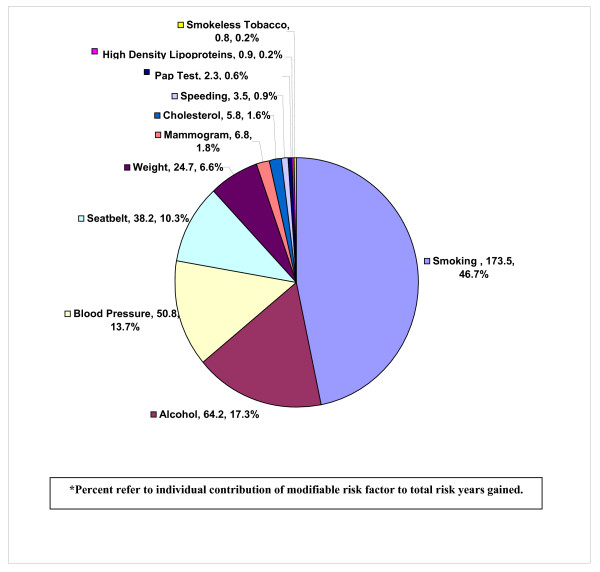
Number and Percent* of Potential Risk Years Gained by Modifiable Risk Factor in our Free Clinic Population.

The total sum of potential risk years gained in the free clinic population of 186 subjects was 371.4. Frequency distributions on potential risk years gained by each of the eleven modifiable risk factors revealed the following, in order of impact: quitting smoking could result in a total of 173.5 risk years gained; reducing alcohol consumption, 64.2 years gained; reducing blood pressure, 50.8 years gained; increasing seatbelt use, 38.2 years gained; weight reduction, 24.7 years gained; having regular mammograms, 6.8 years gained; reducing cholesterol levels, 5.8 years gained; reducing frequency of speeding while driving, 3.5 years gained; having regular pap tests, 2.3 years gained; improving HDL levels, 0.9 years gained; and reducing use of smokeless tobacco, 0.8 years gained (Figure 2).

## Discussion

Health risk assessments provide subjects with individualized feedback on how lifestyle choices impact health and well-being. While the use of this technique as an assessment device for evaluating the effects of health programs is uncertain, its role in contributing to belief and behavior change is better appreciated [8]. This was consistent with the feedback we received from the subjects participating in our Healthy Friday Clinic. Many were pleased with the specificity of the feedback and the educational and community resources provided to help them start making changes.

There are a number of health risk appraisals available to suit a wide variety of needs and budgets [17]. In comparing products, one should insure that algorithms used to generate the risk assessments are updated on a regular basis and valid for use in the population of interest. We were fortunate to partner with the Healthier People Network (HPN), a company that does just that. Obtaining our appraisals and evaluation software at no cost made it possible to use it liberally in our free clinic setting. Although the printouts generated by this package were not user friendly for our patient population, generating personal letters interpreting the information added a level of intimacy to our interaction and was well-received by the Healthy Friday Clinic subjects.

Summing the information on potential risk years gained in this population also helped our free clinic decide what types of health initiatives it would invest in. Since 46.7% of the 371.4 potential risk years gained in this study population were attributed to smoking, the clinic felt well justified in allocating resources for a smoking cessation program.

Alcohol consumption had the second largest impact on risk years gained (17.3%) and taken together with the smoking issue, sensitized the clinic to issues of drug use. Over the course of several months, a number of drug seeking patients as well as several high profile incidents in the community led to an appreciation of a prevalent drug addiction issue in the community. A coordinated community response was undertaken with our free clinic co-sponsoring an educational event for local health care providers on recognizing and treating drug abuse.

Elevated blood pressure and seat belt use were our third and fourth largest contributing factors (13.7% and 10.3% respectively). Since blood pressure was routinely checked on all patients coming into the clinic, this information was interpreted as a need for tighter medical management of the matter. Educational posters on proper automobile safety restraints were hung in the clinic and treatment rooms, and an effort was made to ask subjects about their seatbelt use to create heightened awareness about the issue.

It was interesting to note that while 21.5% of our population was overweight and 48.4% were obese, weight was responsible for only 6.6% of the total number of potential risk years gained. This may reflect the younger age distribution of our population and the fact that added weight has a greater impact on health outcomes such as heart disease and diabetes as one grows older. Regardless, the clinic staff counseled on weight more frequently and made information on programs like Weight Watchers™ more accessible.

All of these factors, taken together with other initiatives that were part of a larger community project, played a role in changing the environment at the free clinic. While we were not able to quantify it, the change was noticeable to staff and patients alike. This pleased us because the free clinic serves a predominantly middle-aged, high school educated, working, uninsured population who usually seeks care only for acute medical needs. We hope that facilitating and embracing a broader medical paradigm that values disease prevention and health promotion will influence the how this population perceives their health and well-being, and empower them to make important lifestyle changes.

## Competing interests

The author(s) declare that they have no competing interests.

## Authors' contributions

PDS conceived the project, provided oversight for its implementation, consulted on the analysis and helped draft the manuscript.

CW helped shape the focus of the manuscript, provided the cleaning and analysis of the data and helped draft the manuscript.

Both authors read and approved the final manuscript.
